# Disruption of endosomal trafficking with EGA alters TLR9 cytokine response in human plasmacytoid dendritic cells

**DOI:** 10.3389/fimmu.2023.1144127

**Published:** 2023-03-20

**Authors:** Matthew J. Wiest, Chao Gu, Hyoungjun Ham, Laurent Gorvel, Mira T. Keddis, Leroy W. Griffing, HyeMee Joo, Jean-Pierre Gorvel, Daniel D. Billadeau, SangKon Oh

**Affiliations:** ^1^ Department of Immunology, Mayo Clinic, Scottsdale, AZ, United States; ^2^ Baylor Institute of Biomedical Studies, Baylor University, Waco, TX, United States; ^3^ Department of Immunology, Mayo Clinic, Rochester, MN, United States; ^4^ CRCM, Aix Marseille Universite, INSERM, Marseille, France; ^5^ Department of Nephrology, Mayo Clinic, Scottsdale, AZ, United States; ^6^ Department of Rheumatology, Mayo Clinic, Scottsdale, AZ, United States; ^7^ CIML, Aix Marseille Universite, INSERM, Marseille, France

**Keywords:** EGA, endosomal trafficking, nucleic acid, plasmacytoid dendritic cells, toll-like receptor 9, type 1 interferon, pro-inflammatory cytokine

## Abstract

Plasmacytoid dendritic cells (pDCs) exhibit bifurcated cytokine responses to TLR9 agonists, an IRF7-mediated type 1 IFN response or a pro-inflammatory cytokine response *via* the activation of NF-κB. This bifurcated response has been hypothesized to result from either distinct signaling endosomes or endo-lysosomal trafficking delay of TLR9 agonists allowing for autocrine signaling to affect outcomes. Utilizing the late endosome trafficking inhibitor, EGA, we assessed the bifurcated cytokine responses of pDCs to TLR9 stimulation. EGA treatment of pDCs diminished both IFNα and pro-inflammatory cytokine expression induced by CpG DNAs (D- and K-type), CpG-DNAs complexed with DOTAP, and genomic DNAs complexed with LL37. Mechanistically, EGA suppressed phosphorylation of IKKα/β, STAT1, Akt, and p38, and decreased colocalization of CpG oligodeoxynucleotides with LAMP^+^ endo-lysosomes. EGA also diminished type 1 IFN expression by pDCs from systemic lupus erythematosus patients. Therefore, our findings help understand mechanisms for the bifurcated cytokine responses by pDCs and support future examination of the potential benefit of EGA in treating type 1 IFN-associated inflammatory diseases in the future.

## 1 Introduction

Plasmacytoid dendritic cells (pDCs) are well characterized for their ability to express type 1 interferon (IFN) in response to nucleic acids derived from self (1–4) and non-self-origins (5–8). In response to certain types of nucleic acids, pDCs can also produce large quantities of pro-inflammatory cytokines, including tumor necrosis factor alpha (TNFα) and interleukin-6 (IL-6) with a minimum amount of type 1 IFN (9, 10).

In consideration of the critical roles of cytokines produced by pDCs in the pathogenesis of certain types of inflammatory diseases, including systemic lupus erythematosus (SLE) (11, 12), it is important to understand cellular and molecular mechanisms for the bifurcated cytokine responses by pDCs (13, 14). In addition, strategies that can allow us to effectively manipulate cytokine expression by pDCs are of potential benefit in controlling auto-inflammatory diseases (11, 12, 15).

Of different types of toll-like receptor 9 (TLR9) agonists, D-type CpG-ODNs (e.g., CpG-ODN2216) and genomic DNA-anti-microbial peptide (e.g., LL37) complexes are noted to induce high levels of type 1 IFN with moderate amounts of pro-inflammatory cytokines (4, 14, 16). In contrast, K-type CpG-ODNs (e.g., CpG-ODN2006) induce pDCs to express pro-inflammatory cytokines and pDC maturation (9, 14, 16). Such bifurcated cytokine responses to different types of nucleic acids has been partly explained with the presence of distinct signaling endosomes within pDCs; an IFN regulatory factor-7 (IRF7) signaling endosome responsible for type 1 IFN production and nuclear factor kappa-light-chain-enhancer of activated B cells (NF-κB) signaling endosome responsible for pro-inflammatory cytokine production and pDC maturation (14, 17, 18). This was further demonstrated by altering the structure of the CpG-ODNs (14, 18) or complexation with peptides or liposomes (4, 13, 19) which alters their endosomal trafficking retention times.

However, the identity markers of reported signaling endosomes still remains to be adequately resolved with differing reports utilizing different cell types, including pDCs (14, 17, 20) and other immune cells (13, 21). Previous studies (13, 14) showed that retention of CpG-ODNs within early/recycling endosome compartments is necessary for the induction of type 1 IFN *via* IRF7 in human pDCs (14) and mouse dendritic cells (13) while other reports have suggested that IRF7 signaling emanates from a vesicle associated membrane 3 (VAMP3)^+^ lysosomal associated membrane protein 2 (LAMP2)^+^ lysosomal related organelle in human pDCs, pDC-like cell lines (17) and mouse cells (21). Furthermore, TLR9-mediated NF-κB signaling has been suggested to emanate from either the late endo-lysosomal compartment in human pDCs (14, 17) or from an early/recycling endosome in mouse bone marrow derived dendritic cells and macrophages (21). Data from all these studies (13, 14, 17, 20, 21) also suggested that the transition between early/recycling to endo-lysosomes could be a key biological step that could determine the bifurcated cytokine response by TLR9 agonists in pDCs.

EGA, 4-bromobenzaldehyde *N*-(2,6-dimethylphenyl) semicarbazone, was first reported as an inhibitor of anthrax lethal toxin-induced pyroptosis in macrophages (22). EGA is known to block the trafficking from early endosome antigen 1 (EEA1)^+^ endosomes to lysosome-associated membrane protein 1 (LAMP1)^+^ endosome/lysosome without affecting recycling endosome trafficking. EGA did not alter the acidification of endo-lysosomes, phago-lysosomal trafficking, and phagosome permeabilization (22).

Therefore, we hypothesized that inhibition of endosomal trafficking from the early/recycling endosome to the endo-lysosome compartment with EGA could alter the bifurcated cytokine responses by pDCs stimulated with TLR9 agonists. We tested this hypothesis by assessing cytokine expression by pDCs in response to two different types of CpG DNAs and genomic DNAs (gDNAs) complexed with an anti-microbial peptide, LL37. Mechanistic insight into the EGA-mediated modulation of bifurcated cytokine responses by pDCs was also investigated. We also tested whether EGA, in comparison with phosphatidylinositol 3-phosphate 5-kinase (PIKfyve) inhibitor, could also alter the cytokine expression by pDCs in the blood of adult SLE patients. EGA treatment of pDCs inhibited TLR9 agonist trafficking to LAMP^+^ endo-lysosomes resulting in diminished cytokine responses to a variety of TLR9 agonist suggesting that TLR9 signals from this compartment.

## 2 Materials and methods

### 2.1 pDC isolation and culture

Buffy coats (Oklahoma Blood Institute) and apheresis cones from Mayo Clinic were utilized as blood sources. Peripheral blood mononuclear cells (PBMC) were collected from the blood by density gradient centrifugation utilizing Ficoll-Paque Plus (GE Healthcare). DCs were enriched from PBMCs with Pan-DC Enrichment Kit (Stemcell Technologies. pDCs (Lin-1^-^, HLA-DR^+^, CD123^+^, CD11c^-^) were sorted on a fluorescence-activated cell sorting (FACS) Aria II (BD Biosciences). Purity after sorting was above 98%. pDCs were washed with complete RPMI (cRPMI), counted, and resuspended in cRPMI/10% fetal bovine serum (FBS; Gemini Bio-Products) containing 5ng/mL IL-3 (R&D Systems). cRPMI consisted of RPMI1640 containing L-glutamine (ThermoFisher) supplemented with MEM non-essential amino acids, sodium pyruvate (Sigma Aldrich), and penicillin/streptomycin (ThermoFisher).

YM201636 (PIKfyve inhibitor; Cayman Chemical) and EGA (Cayman Chemical or Sigma Aldrich) were dissolved in dimethyl sulfoxide (DMSO; ThermoFisher), aliquoted, and frozen at -30°C. Fresh aliquots were utilized for each experiment. Cells were pre-incubated for at least 15-30 minutes with 1 μM YM201636 or 20 μM EGA at 37°C. Approximately 5x10^4^ cells were stimulated with 2 μg/mL ODN2216, 2 μg/mL ODN2006 purchased from Invivogen, or 2 multiplicity of infection (MOI) live influenza A PR8/34 (Provided by Adolfo Garcia-Sastre, Mount Sinai, NY). Complexes of 10 μg/mL genomic DNA from spleen (Biochain) with 50 μg/mL LL-37 (Innovagen) and 2 μg/mL CpG-ODN with 10 μg/mL 1,2-Dioleoyl-3-trimethylammonium propane (DOTAP Transfection Reagent; Santa Cruz Biotechnology) were prepared in LAL water (Invivogen) at least fifteen minutes prior to cell stimulation. Cells were stimulated for two hours and fourteen hours upon which GolgiPlug (BD Biosciences) was added according to the manufacturer’s instructions. Three and four hours later respectively, cells were washed with 2 mM EDTA/Dulbecco’s phosphate buffered saline (DPBS; ThermoFisher) and stained for viability, fixed and permeabilized with Cytofix/CytoPerm (BD Biosciences), and finally stained for intracellular cytokines and isotype controls in PermWash solution (BD Biosciences).

Titration and kinetic studies were performed in a similar setup with the exceptions of differences in dosage of EGA and pre-incubation time points. Kinetic studies were performed with 1 μg/mL CpG-ODN2216 due to high variability in response between donors at higher dosages.

### 2.2 Flow cytometry and antibodies

**Supplementary Table 1** lists the antibodies utilized for FACS analysis in this study. Cells were run on a FACS Celesta (BD Biosciences). Flow cytometry data were analyzed utilizing FlowJo v10 (FlowJo). Intracellular cytokine staining gating was based on the examination of cytokine staining for unstimulated cells and isotype control staining.

### 2.3 Antibody-coated bead-based multiplex cytokine assay

Bead-based Luminex kits assessing human IFNα2, IL-6, and TNFα from ThermoFisher were utilized in assessing cytokine concentrations in supernatants from overnight stimulated blood pDCs according to the manufacturer’s instructions. Beads were assessed on a Luminex 200 machine and the Xponent 3.1 software (Luminex) was utilized to generate a five-parameter logistic regression standard curve from which cytokine levels were determined. Technical replicates were averaged before analysis with statistical tests.

### 2.4 Immunoblotting

After sorting, pDCs were extensively washed and rested for at least one hour in serum-free cRPMI containing IL-3 (5 ng/mL) at 37 °C. After one hour, indicated inhibitors were added to pDCs and placed at 37 °C for 30 minutes. Approximately 1x10^5^ - 1.4x10^5^ pDCs/well in 96-well plates were then stimulated with 1 μM ODN2216 for the appropriate time frame upon cell lysates were then harvested in RIPA buffer (ThermoFisher) supplemented with HALT™ protease and phosphatase inhibitor cocktail (ThermoFisher). pDCs from multiple donors were combined at the cell lysate harvest stage. Cell lysates were reduced and separated on Novex 4 to 20% Tris-Glycine gels (Invitrogen) and transferred to PVDF membranes (Bio-Rad) as previously described (23). Protein concentration in each lane was then determined utilizing No-Stain Protein labeling reagent (ThermoFisher) according to the manufacturer’s instructions before antibody probing. Antibodies utilized for immunoblotting assay in this study are listed in **Supplementary Table 2**. PageRuler pre-stained protein ladder (ThermoFisher, Catalog 26616) was used as a reference for protein size. Images were acquired on a ChemiDoc MP (Bio-Rad) and analyzed in Image Lab V6.0 (Bio-Rad). Protein intensity was normalized either to its reference protein or total protein concentration and relative intensity was obtained by comparison with the vehicle-treated group at time zero.

### 2.5 Lysotracker staining

After sorting, pDCs were extensively washed and resuspended in cRPMI/10% FBS containing IL-3 (5 ng/mL). pDCs were pre-incubated with 20 μM EGA, 1 μM YM201636, 1 μM Bafilomycin A1 (Invivogen), or DMSO for 1 hour at 37 °C. Approximately 1x10^5^ - 1.25x10^5^ pDCs/well in 96-well plates were then stimulated with 2 μg/mL CpG-ODN2216 for 12 hours. At 12 hours, pDC were washed and stained with LysoTracker Deep Red (ThermoFisher). For Lysotracker staining, cells were washed and stained with 50 nM LysoTracker in cRPMI/10% FBS for 30 minutes at 37 °C. Cells were then washed with ice-cold DPBS, stained for viability on ice and then run on FACS Fortessa (BD).

### 2.6 Confocal microscopy

pDCs were enriched utilizing a similar procedure as described previously except for a switch to a plasmacytoid dendritic cell enrichment kit (Stemcell Technologies). Enriched cells were assessed for purity utilizing the same panel utilized for sorting. pDC purity was above 85%. pDCs were pre-incubated with inhibitors for 1 hour at 37°C before the addition of 1μM ODN2216-FITC. Cells were washed extensively 20 minutes before the indicated time point in pre-warmed media with inhibitors and then allowed to attach to poly-D-lysine-coated (35 μg/mL; ThermoFisher) coverslips for 10 minutes. Cells attached to coverslips were then stained in a manner as previously described (24). Briefly, pDCs were fixed with ice-cold 4% paraformaldehyde (Electron Microscopy Sciences) for 18 minutes. Cells were permeabilized with 0.15% Triton™ X-100 Surfactant-Amps (ThermoFisher) for 3 minutes and then washed extensively. Cells were blocked and stained in a saline blocking buffer containing 5% goat serum (Invitrogen), 1% glycerol (Sigma-Aldrich), and 0.1% bovine serum albumin (MP Biomedicals). After blocking for 1 hour, cells were incubated with primary and secondary antibodies in blocking buffer with extensive washing between steps. Cells were stained with antibodies listed in **Supplementary Table 3**. Cells were then counterstained with Hoechst 33342 (ThermoFisher) according to the manufacturer’s instructions and washed. Prolong Gold Antifade Mountant reagent (ThermoFisher) was added to slides and coverslips were mounted and allowed to cure overnight. Slides were then imaged on an LSM 800 Confocal Microscope (Zeiss) utilizing a 63x/1.4N.A objective. Identical acquisition settings were used for all experimental samples and images were scanned in frame mode to avoid any crosstalk between fluorophore signals.

### 2.7 Image processing

Cell images were Airyscan processed in the Zen software module (Zeiss). Images were further processed in the open-source software Fiji (25). Baseline intensity thresholds of endo-lysosomal markers for analysis were defined by cellular autofluorescence and isotype antibody intensity staining for each experiment. Manders’ coefficients were determined utilizing an in-house customized plugin coded for thresholding of ODN signal and region of interest selection.

### 2.8 PBMC isolation and culture

Systemic lupus erythematosus patients (**Supplementary Table 4**) were recruited at Mayo Clinic Scottsdale under a plan approved by the Institutional Review Board (18-011501). Patients provided informed consent in accordance with the Declaration of Helsinki. PBMCs were isolated from the whole blood of SLE patients. PBMCs were washed in cRPMI and resuspended in cRPMI/10% FBS (Gemini Bio-Products) and pre-incubated with 1 μM YM201636, for 30 minutes at 37°C or 20 μM EGA or DMSO for 15 minutes at 37°C. Approximately 8x10^5^ - 1x10^6^ PBMCs were stimulated with 2 μg/mL CpG-ODN2006 complexed with 10 μg/mL DOTAP Transfection Reagent (Santa Cruz Biotechnology), or 2 MOI PR8 influenza virus in 48-well plates. After 2 hours of incubation, GolgiPlug (BD Biosciences) was added according to the manufacturer’s instructions. Three hours later cells were stained for viability and surface markers. Cells were fixed and permeabilized with Cytofix/CytoPerm (BD Biosciences), and finally stained for intracellular cytokine expression. Cells were run on FACS Fortessa (BD Biosciences), and data were analyzed utilizing FlowJo v10 (FlowJo).

### 2.9 Data and statistical analysis

Graphs with error bars represent mean ± SD. Data were analyzed in Prism 7.0 (Graphpad) utilizing paired t-test, one-way analysis of variance (ANOVA) and two-way ANOVA with Tukey, Sidak, or Dunnett’s *post-hoc* multiple comparisons test. Significance was set at p<0.05.

## 3 Results

### 3.1 EGA can modify cytokine response of pDCs to TLR9 stimulation

We first tested whether EGA could affect pDC responses to synthetic CpG DNAs, CpG-ODN2216 (D-type) and CpG-ODN2006 (K-type). Purified pDCs from the blood of healthy subjects were pre-treated with 20 μM EGA before stimulation with indicated CpG-ODNs. We also utilized influenza A, which requires processing in both the early and late endosomes for viral uncoating (26), as an additional stimulus.

**Figure 1:**
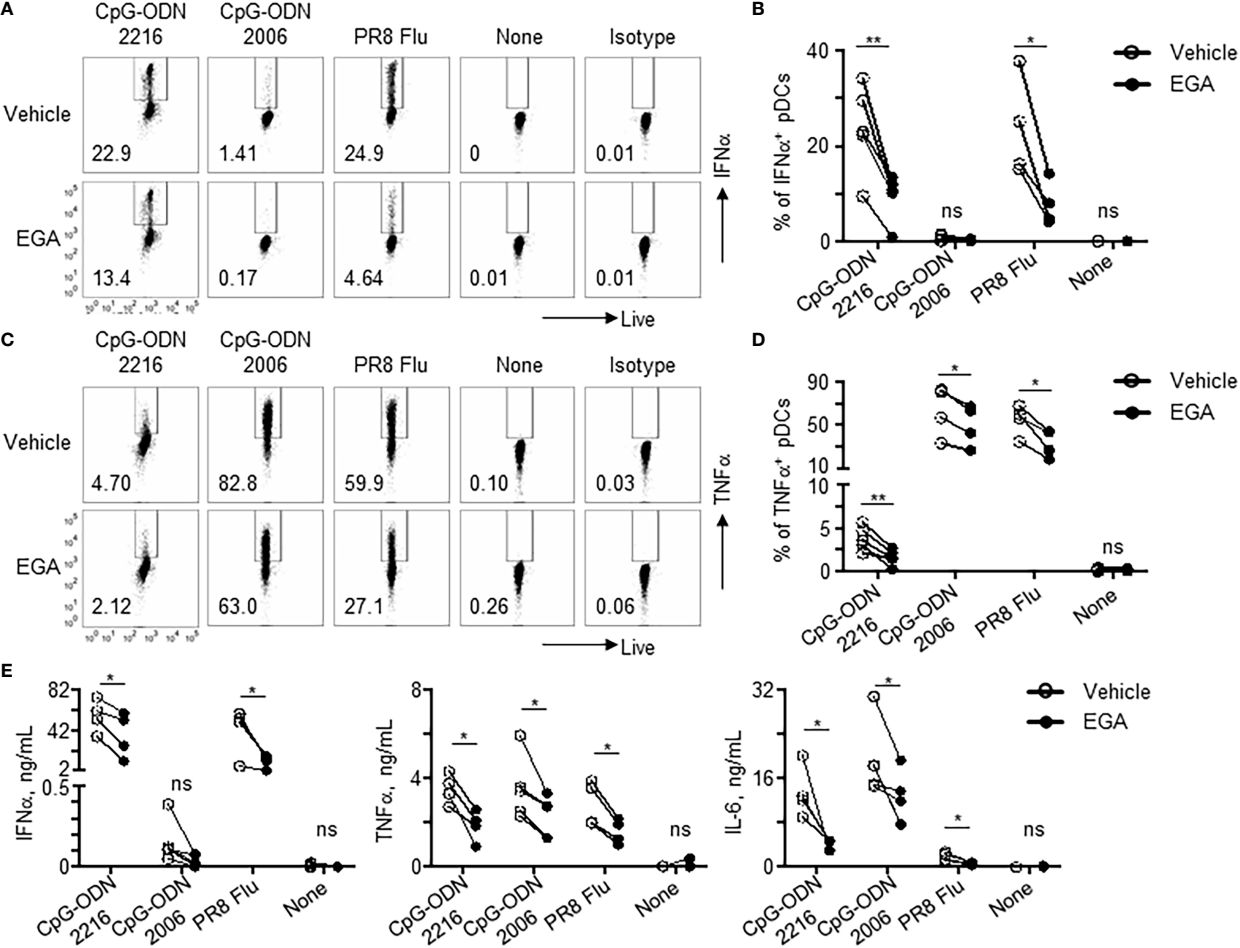
EGA diminishes cytokine expression in pDCs stimulated with CpG-ODNs and influenza virus. **(A–D)** pDCs pre-incubated with 20 μM EGA or vehicle, stimulated with 2 MOI PR8 influenza virus (A/PR8, H1N1) or CpG-ODN2006 for 5 hours or CpG-ODN2216 overnight and stained for IFNα **(A, B)** and TNFα (**C**, **D**). Representative flow cytometry plots **(A, C)** and compiled data set **(B, D)** from 4-5 different subjects. **(E)** Cytokines in supernatants of pDCs pre-incubated with 20 μM EGA or vehicle and stimulated with 2 MOI PR8 influenza virus (A/PR8, H1N1) or TLR9 agonists overnight. Compiled data from 4-5 different subjects. Data analyzed by two-tailed paired t-test. *P< 0.05, **P< 0.01, ns, not significant, for comparison between groups.

As shown in **Figure 1**, EGA-treated pDCs showed diminished responses to TLR9 stimulation, as assessed by measuring the frequency of pDCs expressing IFNα and TNFα. EGA pre-treatment decreased IFNα expression by CpG-ODN2216-treated pDCs (**Figures 1A, B**). EGA also decreased TNFα expression in CpG-ODN2216- and CpG-ODN2006-treated pDCs (**Figures 1C, D**). EGA could also effectively diminish pDC responses to influenza A virus (**Figures 1A–D**). The amount of cytokines (IFNα, TNFα, and IL-6) in culture supernatants (**Figure 1E**) of pDCs stimulated overnight with CpG-ODNs further confirmed the intracellular cytokine expression data in **Figures 1A–D**. Overall, data in **Figure 1** indicate that EGA can suppress the expression of both IFNα and proinflammatory cytokines by pDCs stimulated with both types of CpG-ODNs.

**Figure 2:**
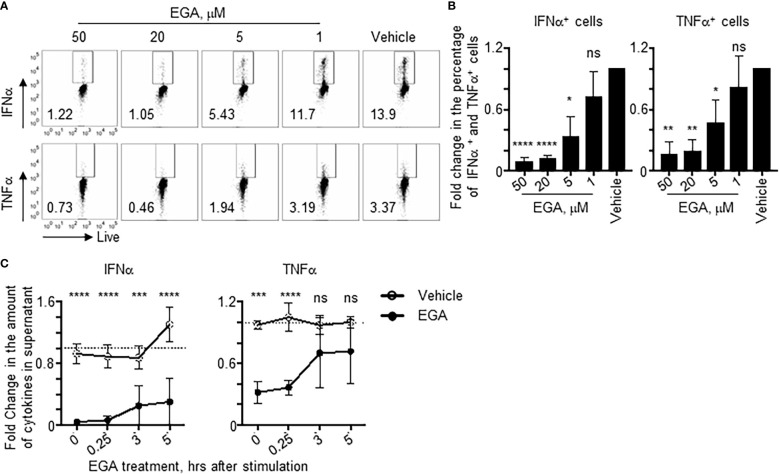
Kinetics of EGA-mediated suppression of cytokine expression in pDCs stimulated with CpG-ODNs. **(A, B)** pDCs pre-incubated with indicated concentrations of EGA before stimulation with CpG-ODN2216. Representative flow cytometry plots **(A)** and compiled data of fold change relative to vehicle treated cells **(B)** after overnight stimulation from 4 different subjects. **(C)** pDCs stimulated with CpG-ODN2216 overnight with 20 μM EGA or vehicle added at time points post-CpG-ODN2216 stimulation. Fold changes in the supernatant cytokine concentration after overnight stimulation based on averaging concentration from all time points in vehicle treated cells as reference point. Data from 4 different subjects. Data analyzed by **(B)** 1-way ANOVA followed by Dunnett’s multiple comparison or **(C)** 2-way ANOVA followed by Sidak’s multiple comparison test. *P< 0.05, **P< 0.01, ***P< 0.001, ****P<0.0001, ns, not significant, for comparison between groups.

We next examined the titration effects and kinetics of EGA in the suppression of IFNα and TNFα expression by pDCs stimulated with CpG-ODN2216, which induces both IFNα and TNFα expression (**Figure 2**). EGA showed similar concentration efficacy as outlined in previous work (22), with 20 μM pre-treatment abrogating both IFNα and TNFα expression effectively with lower doses having less of an impact (**Figures 2A, B**). We also examined the kinetics of the cytokine response and the effectiveness of EGA (20μM) in altering cytokine expression by measuring the amount of IFNα and TNFα in culture supernatant after overnight stimulation of pDCs with CpG-ODN2216. The addition of EGA at 5 hours post-CpG-ODN2216 stimulation still resulted in a significant reduction in IFNα secretion by pDCs (**Figure 2C**, left). In contrast, EGA was ineffective at reducing TNFα secretion when added to culture 3 hours post-ODN2216 stimulation when normalized to each donor (**Figure 2C**, right). The actual amount of cytokine values used in **Figure 2C** are presented in **Supplementary Figure 1**. Taken together, data in **Figure 2** suggest that EGA can diminish pDC cytokine response by blocking CpG-ODN access to TLR9 activating endosomes, but its effects on the activation of NF-κB and IRF7 signaling pathways might not be the same.

### 3.2 EGA modifies TLR9 response of pDCs to CpG-DNA/DOTAP and gDNA/LL37 complexes

**Figure 3:**
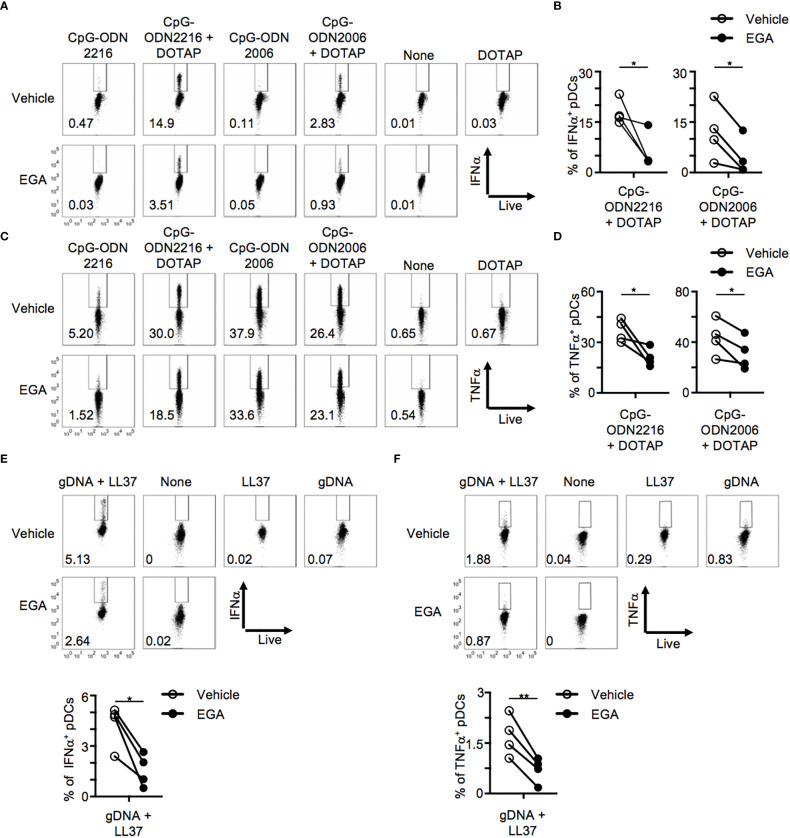
EGA affects cytokine production in CpG-ODN/DOTAP- and genomic DNA/LL37- stimulated pDCs. **(A–D)** pDCs pre-incubated with 20 μM EGA or vehicle, stimulated with free CpG-ODN or complexed to DOTAP for 5 hours and stained for IFNα and TNFα. Representative flow cytometry plots from one experiment **(A, C)** and compiled data **(B–D)** from 4 different subjects. **(E, F)** Blood pDCs pre-incubated with 20 μM EGA or vehicle, stimulated with genomic DNA and LL37 overnight, and stained for IFNα **(E)** and TNFα **(F)**. Representative flow cytometry plots and compiled data from 4 different subjects. Data analyzed by two-tailed paired t-test. *P< 0.05, **P< 0.01, for comparison between groups.

Complexation of CpG-ODN with the liposomal reagent, DOTAP, alters the cytokine response of macrophages and dendritic cells with CpG-ODN2006 complexed to DOTAP inducing increased type 1 IFN expression (13, 19). Mechanistically, DOTAP complexation enhances CpG-ODN uptake amount and kinetics of uptake as free CpG-ODN is highly saturable (19, 27). Furthermore, it has been shown that forming multimeric structures by complexing CpG-ODN to DOTAP may alter their endo-lysosomal trafficking (13, 19). We thus examined whether EGA would also be effective in diminishing the cytokine expression of pDCs in response to CpG-ODNs complexed with DOTAP. Complexing of CpG-ODNs, both ODN2216 and ODN2006, to DOTAP enhanced IFNα expression (**Figure 3A**, top), as previously described (19). IFNα was detectable at 5 hours post-stimulation when CpG-ODNs were complexed with DOTAP which was not seen with free CpG-ODNs (**Figure 3A**), which required overnight stimulation in the case of CpG-ODN2216 stimulation (**Figure 1**). Notably, EGA was still effective at diminishing IFNα (**Figures 3A, B**) and TNFα (**Figures 3C, D**) expression induced by CpG-ODN2216/DOTAP and CpG-ODN2006/DOTAP. In line with the decreased frequency of IFNα^+^ and TNFα^+^ pDCs, EGA treatment decreased the amount of IFNα and TNFα secretion by pDCs stimulated with CpG-ODN2216 or CpG-ODN2216/DOTAP (**Supplementary Figure 2A**) as well as pDCs stimulated with CpG-ODN2006 or CpG-ODN2006/DOTAP (**Supplementary Figure 2B**).

The antimicrobial peptide, LL-37, has been implicated in the breaking of tolerance in psoriasis (4) and SLE (2) through the formation of complexes with self-DNAs which activate pDCs through TLR9 with increased retention in early endocytic compartments (4). We, therefore, assessed whether EGA would also be effective in diminishing pDC response to gDNA/LL-37 complexes. gDNAs and LL-37 were not able to induce cytokine expression individually but when complexed together was able to induce both IFNα and TNFα (**Figures 3E, F**) expression in pDCs. EGA pre-treatment was also capable of suppressing both IFNα and TNFα expression by pDC stimulated with gDNA/LL-37 complexes (**Figures 3E, F**). EGA was capable of diminishing IFNα secretion by gDNA/LL37 complexes (**Supplementary Figure 2C**). Therefore, we concluded EGA treatment results in decreased expression of both IFNα and pro-inflammatory cytokine by pDCs stimulated with synthetic CpG DNAs and IFNα expression induced by gDNA/LL37 complexes which are implicated in autoimmune conditions (2, 4).

### 3.3 EGA and PIKfyve inhibitor, YM201636, can diminish pDC cytokine expression

A previous report has suggested that abrogation of phosphatidylinositol 3-phosphate 5-kinase (PIKfyve) activity impedes the trafficking of ODNs to the late endosome and lysosome, which is associated with TLR9 activation (28), in primary murine macrophages and cell line, RAW 264.7 (29). We, therefore, compared a PIKfyve inhibitor, YM201636, with EGA for their ability to modify cytokine expression by pDCs stimulated with CpG-ODN2216.

**Figure 4:**
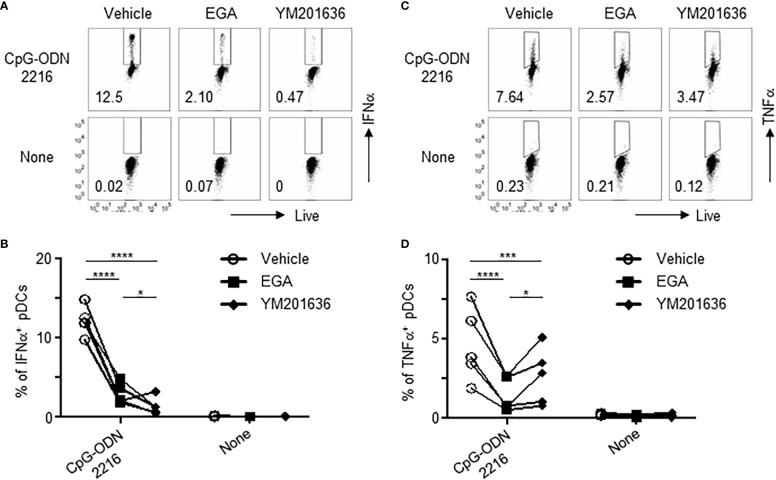
EGA and PIKfyve inhibitor, YM201636, can reduce IFNα and TNFα expression by pDCs treated with CpG-ODN2216. **(A–D)** pDCs pre-incubated with 20 μM EGA, 1 μM YM201636, or vehicle, stimulated with CpG-ODN2216 overnight, and stained for IFNα and TNFα. Representative flow cytometry plots and compiled data for IFNα **(A, B)** and TNFα **(C, D)** from 5 different subjects. Data analyzed by 2-way ANOVA followed by Tukey multiple comparison test. *P< 0.05, ***P< 0.001, ****P<0.0001, for comparison between groups.

Both EGA and YM201636 reduced IFNα (**Figures 4A, B**) and TNFα (**Figures 4C, D**) expression in CpG-ODN2216-stimulated pDCs. The amount of IFNα and TNFα in the culture supernatants (**Supplementary Figure 3**) was consistent with the frequency of IFNα+ and TNFα+ pDCs (**Figure 4**). We also found that YM202636 was more effective than EGA at inhibiting IFNα expression, as determined by both the frequency of IFNα^+^ pDCs (**Figure 4B**) and the amount of IFNα secreted in the culture supernatants (**Supplementary Figure 3**). Although EGA was more effective than YM201636 at decreasing the frequency of TNFα^+^ pDCs (**Figure 4D**), such difference between the two inhibitors was not observed when the amount of TNFα in the culture supernatant was assessed after overnight stimulation (**Supplementary Figure 3**). EGA and YM201636 used in this study did not alter pDC viability after overnight stimulation (**Supplementary Figure 4**). Therefore, we concluded that both endosomal trafficking inhibitors, EGA and YM201636, could decrease both IFNα and TNFα expression by CpG-ODN2216-stimulated pDCs. Our data also suggested that the two inhibitors might not be the same at suppressing IRF7-mediated IFNα expression and NF-κB-mediated TNFα expression.

### 3.4 EGA and YM201636 treatment can differentially affect the activation of kinases related to endosomal trafficking and cytokine expression

We further investigated mechanisms of action of the two endosomal trafficking inhibitors, EGA and YM201636, in regulating cytokine expression by CpG-ODN-stimulated pDCs by assessing the activation levels of several kinases that are involved in endosomal trafficking and CpG-ODN-induced cytokine expression. These include p38 activation for TLR9 downstream signaling in the IRF7 (30) and NF-κB pathway (31), Iκβ kinase α (32, 33) and β (34) activation for type 1 IFN expression, STAT1 activation (30) and ATF3 induction (35, 36) for downstream type 1 IFN signaling and ISGF3 complex formation, p65 activation for pro-inflammatory cytokine expression (37), and PI3K/Akt activation in controlling endosome trafficking and vacuolar-ATPase-mediated endosome acidification (38–41).

**Figure 5:**
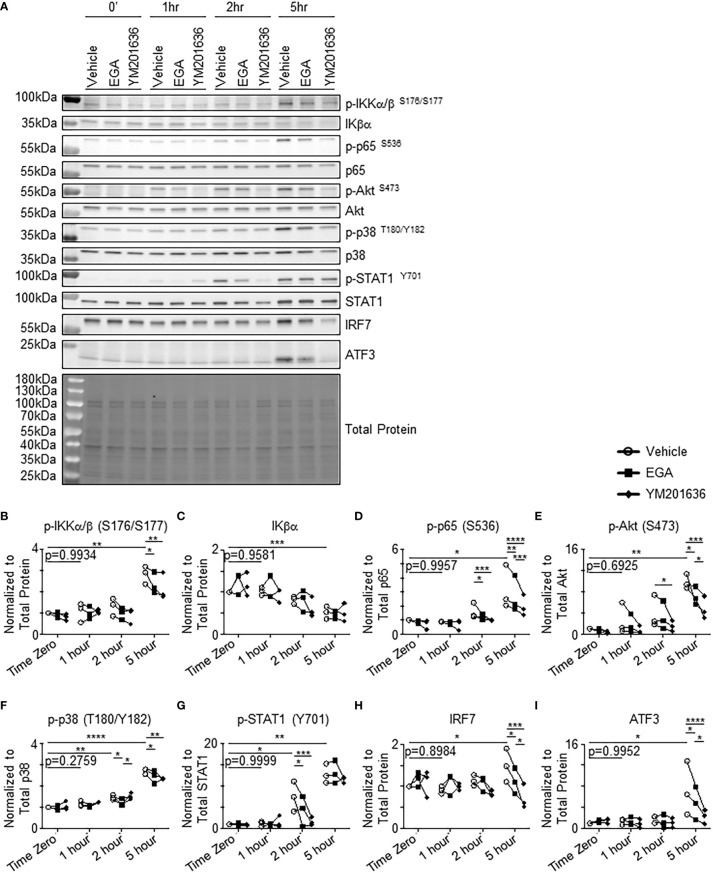
EGA and YM201636 differentially inhibit activation of kinases involved in endosomal trafficking and CpG-ODN-mediated cytokine expression. **(A–I)** pDCs pre-incubated with 20 μM EGA, 1 μM YM201636 or vehicle and stimulated with 1 μM CpG-ODN2216 for indicated time. Representative blot **(A)** with summarized quantified data changes in kinases **(B–I)**. Representative of 3 independent experiments with kinase kinetics analyzed with vehicle-treated group by one-way ANOVA with Dunnett’s multiple comparison test. Inhibitor data analyzed by two-way ANOVA with Tukey multiple comparison test. *P< 0.05, **P< 0.01, ***P< 0.001, ****P<0.0001, for comparison between groups.

Similar to the previously published data (31), low levels of phosphorylation of IKKα/β, p65, STAT1, and p38 were detectable before stimulation (**Figure 5A**). Previous studies also reported that pDCs express tonic levels of type 1 interferon which has autocrine effects on the basal levels of pDC activation (42, 43). Increases in phosphorylation of IKKα/β (**Figure 5B**) were observed at 5 hours post-stimulation with decreases of IKKα/β activation similarly by EGA and YM201636. The amount of total IKβα was reduced at 5 hours post-stimulation (**Figure 5C**). Phosphorylation of p65 was also increased at 5 hours (**Figure 5D**). Such an increase of p65 activation was suppressed by both inhibitors, but YM201636 was more effective than EGA. Enhanced phosphorylation of Akt (**Figure 5E**) and p38 (**Figure 5F**) was also observed at 5 and 2 hours after stimulation, respectively. There was no significant increase of the activation of IKKα/β, p65, or p38 between time “0” and “1 hour” after activation. It was also of note that YM201636 was more effective than EGA at lowering Akt activation levels at 5 hours post-stimulation, whereas EGA was more effective than YM201636 at lowering p38 activation levels at 2 hours post-activation. CpG-ODN2216 also induced STAT1 phosphorylation (**Figure 5G**) (30), as well as increased expression of both IRF7 (**Figure 5H**) and ATF3 (**Figure 5I**), a type 1 interferon-inducible transcription factor, which can repress type 1 interferon and cytokine responses (35, 36). IRF7 protein expression in human pDCs was previously reported to be upregulated in CpG-ODN-stimulated cells beginning at 3 to 5 hours (30, 44, 45) which is responsible for the production of type 1 interferon induced by CpG-ODN. STAT1 phosphorylation was also lowered by both EGA and YM201636 (**Figure 5G**) starting at 2 hours, indicative of decreased type 1 IFN expression which depressed IRF7 (**Figure 5H**) enhancement at 5 hours and affected ATF3 induction (**Figure 5I**). In support of the data in **Figure 4B**, in which YM201636 was more effective than EGA in suppressing IFNα expression, YM201636 was superior to EGA at decreasing the upregulation of IRF7 and ATF3.

Collectively, EGA or YM201636 treatment could result in altered activation levels of several kinases that are related to endosomal trafficking and cytokine expression by pDCs. Except for the suppression of p38 activation at 2 hours post activation, YM201636 was more effective than EGA at suppressing the activation of all other kinases tested in this study. These results further confirm that EGA and YM201636 suppress CpG-ODN responses within pDCs and also suggest that they inhibit a process that is necessary for TLR9 signaling. Furthermore, EGA and YM201636 treatment both resulted in decreased phosphorylation of Akt (38–41) which is involved in endosome trafficking and acidification suggesting that TLR9 signaling occurs in an endo-lysosomal compartment potentially accessed through the activity of Akt.

### 3.5 EGA and YM201636 block the trafficking of TLR9 agonists

To gain a better insight into cellular mechanisms in which EGA and YM201636 affect cytokine expression by pDCs, we next examined the trafficking of CpG ODNs in pDCs in the presence and absence of EGA or YM201636.

A trace of CpG-ODN2216-fluorescein isothiocyanate (FITC) uptake within pDCs was performed to determine an optimal time frame to analyze its subcellular localization (**Supplementary Figure 5**). At 30 minutes post-stimulation, CpG-ODN2216 was limited to the cell periphery with minimal overlap with endo-lysosomal markers. CpG-ODN2216-FITC had a perinuclear distribution and began to overlap with endo-lysosomal markers starting at 2 hours (**Supplementary Figure 5**).

**Figure 6:**
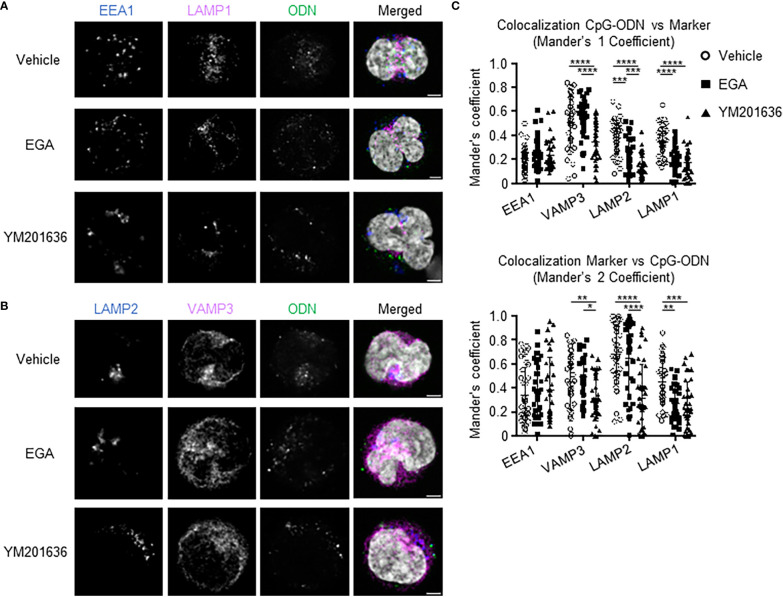
EGA and YM201636 treated pDCs have decreased colocalization of CpG-ODN2216-FITC with late endosome/lysosome markers. **(A–C)** Enriched pDCs pre-incubated with 20 μM EGA, 1μM YM201636 or vehicle for 1 hour before addition of 1 μM CpG-ODN2216-FITC for 2 hours. Representative staining of endo-lysosomal markers and CpG-ODN within pDCs **(A, B)** and summarized colocalization data **(C)** of 38 cells from 3 experiments. Scale bar 2μm. Data analyzed in **(C)** by 2-way ANOVA followed by Tukey multiple comparison test.*P< 0.05, **P< 0.01, ***P < 0.001, ****P<0.0001, for comparison between groups.

We assessed CpG-ODN2216 co-localization at 2 hours within pDCs treated with EGA or YM201636. Overall, CpG-ODN2216 was localized to the cell periphery in EGA- and YM201636-treated pDCs when compared to vehicle-treated (**Figures 6A, B**). Co-localization analysis of CpG-ODN2216 showed that the two inhibitors decreased CpG-ODN2216 trafficking to LAMP1^+^ and LAMP2^+^ compartments (**Figure 6C**, top). It was also of note that YM201636 was more effective than EGA at inhibiting its co-localization with LAMP2^+^ compartments. Moreover, YM201636, but not EGA, also lowered co-localization of CpG-ODN2216 with VAMP3 (**Figure 6C**, top). Analysis of localization of endo-lysosomal markers with CpG-ODN2216 further suggested that EGA and YM201636 blocked the trafficking of CpG-ODN2216 to LAMP1^+^ compartments (**Figure 6C**, bottom). YM201636 also suppressed the colocalization of VAMP3 and LAMP2 with CpG-ODN2216 (**Figure 6C**, bottom), suggesting that YM201636 could potentially suppress the formation of inducible VAMP3^+^LAMP2^+^ compartment in an early activation stage (17). Neither EGA nor YM201636 altered the co-localization of CpG-ODN2216 with EEA1 in early endosomal compartments. **Supplementary Figure 6** presents additional representative images of CpG-ODN2216 with EEA1, LAMP1, LAMP2, and VAMP3.

Therefore, we concluded that EGA and YM201636 both diminished the trafficking of CpG-ODN2216 to LAMP^+^ compartments including the lysosomal-related organelle and lysosome, but their effectiveness was not the same. In contrast to EGA, YM201636 also inhibited CpG-ODN2166 trafficking to VAMP3^+^ compartments including recycling endosomes. In line with the previously reported data (22), EGA did not affect endosome acidification, as measured by assessing the intensity of LysoTracker (**Supplementary Figure 7**), in comparison to Bafilomycin A1 treatment.

### 3.6 EGA affects the cytokine response in SLE pDCs

**Figure 7:**
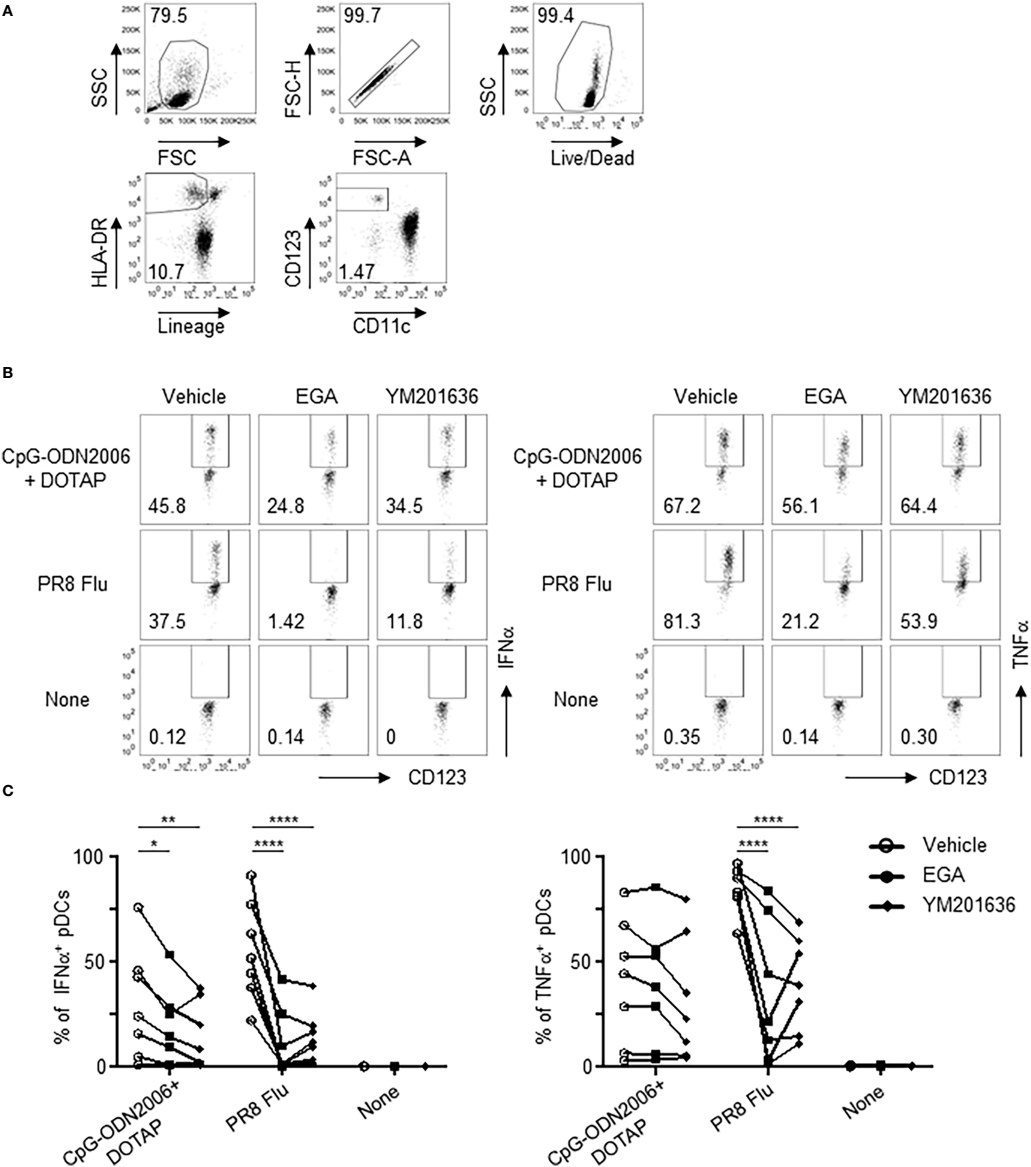
EGA affects TLR7/9 agonist responses in SLE pDCs. **(A–C)** PBMCs from SLE patients pre-incubated with 20 μM EGA, 1 μM YM201636, or vehicle, stimulated with CpG-ODN2006/DOTAP or 2 MOI PR8 influenza virus (A/PR8, H1N1) for 5 hours. Representative flow cytometry gating for pDCs **(A)** and IFNα and TNFα expression **(B)** and compiled data **(C)** from 7 different subjects. Data analyzed in **(C)** by 2-way ANOVA followed by Tukey multiple comparison test.*P< 0.05, **P< 0.01, ****P<0.0001, for comparison between groups.

We next investigated whether EGA and YM201636 could alter IFNα and TNFα expression by pDCs from SLE patients stimulated with TLR9 ligands. SLE is an autoimmune disease characterized by a type 1 IFN signature (46–49) with a possible role for aberrant TLR9 activation in pathogenesis (2, 50). pDCs from SLE patients showed a diminished type 1 IFN response to CpG-ODN2216 stimulation in comparison to pDCs from healthy controls (51). In response to CpG-ODN2006, however, SLE pDCs show similar NF-κB activation to pDCs from healthy subjects (52). However, CpG-ODN2006 cannot efficiently induce type 1 IFN. Therefore, we used CpG-ODN2006/DOTAP complexes that can effectively induce type 1 IFN, as shown in **Figure 3**. Influenza A virus was used as a positive control, to examine the effects of EGA and YM201636 on IFNα and TNFα expression by pDCs derived from SLE patients (**Figure 7A**). DOTAP, a synthetic complexing agent, promotes endosomal uptakes of DNAs similarly to LL37 (19, 53).

SLE pDCs were responsive to CpG-ODN2006/DOTAP complex stimulation after 5 hours (**Figures 7B, C**) albeit with considerable variability. EGA- and YM201636-treatment reduced IFNα expression in CpG-ODN2006/DOTAP-stimulated SLE pDCs (**Figures 7B, C**). Although both EGA- and YM201636-treatment reduced TNFα expression in some of the SLE pDCs stimulated with CpG-ODN2006/DOTAP complexes this was not significant for either treatment (**Figures 7B, C**). Both EGA and YM201636 could also diminish pDC responses to influenza viral infection (**Figures 7B, C**).

We, therefore, concluded, that EGA and YM201636 are capable of suppressing IFNα expression by SLE pDCs stimulated with CpG-ODN2006/DOTAP. However, neither EGA nor YM201636 could suppress CpG-ODN2006/DOTAP-induced TNFα expression by SLE pDCs, which is contrast to pDCs from healthy subjects (**Figures 3C, D**).

## 4 Discussion

TLR9-mediated bifurcated cytokine response in pDCs has been partly explained by the localization of nucleic acid ligands within distinct endosomes: multimeric D-type ODNs and gDNA/LL-37 complexes are retained within early/recycling endosome compartments while monomeric K-type ODNs are localized to late endosome/lysosomes (13, 14). Furthermore, alterations to the structure of CpG-ODNs change their localization and subsequently the pDC response (4, 14). Dissociation of CpG-ODN/DOTAP complexes at specific pH levels also delineated that CpG-ODN must interact with TLR9 at a higher pH than the late endosome for IFNα expression (14). However, recent studies in mouse macrophages and bone marrow dendritic cells (21, 29) and human blood pDCs and pDC cell lines (17) suggest a lysosomal-related organelle (VAMP3^+^LAMP2^+^) is responsible for IRF7 activation. Loss of genes associated with the formation of lysosomal-related organelles also shows diminished TLR9-mediated type 1 interferon responses in mouse pDCs (54). Therefore, the delineation of TLR9 signaling endosomes in human blood pDCs remains to be further examined. This study has extended these findings by investigating how pDCs respond to different types of nucleic acid ligands in the presence and absence of the endosome trafficking inhibitor, EGA (22).

EGA treatment of pDCs diminished their cytokine response to both types of CpG-ODNs in free form and complexed to DOTAP. Furthermore, EGA was effective in diminishing pDC IFNα responses to gDNA/LL37 complexes. When we compared EGA with the PIKfyve inhibitor, YM201636, we noticed a similar effect on cytokine responses when it came to CpG-ODNs, suggesting that inhibiting endosomal trafficking to late endosomal compartments is an effective strategy for diminishing TLR9 activation. However, it was also of note that YM201636 was more effective than EGA at diminishing IFNα expression (**Figure 4**). Such higher potency of YM201636, compared to EGA, on the suppression of IFNα expression could be further supported by its potent ability to suppress the activation of IKKα/β (32–34) and p38 (30, 31), which are further supported by the decreases of STAT1 and ATF3 expression. Furthermore, EGA and YM201636 were effective at diminishing TNFα expression after overnight stimulation supported by their suppression of p65 phosphorylation at 5 hours.

As EGA does not have a known target (22) we also examined kinases implicated in endo-lysosome maturation. We found that both EGA and YM201636 could suppress the phosphorylation of Akt (38–41) which is implicated in the maturation and acidification of the endo-lysosome. However, EGA and YM201636 did not affect endo-lysosomal acidification when compared to bafilomycin A1 treatment. Our data further demonstrated that EGA treatment affected the trafficking process of both types of CpG-ODNs as well as gDNAs within pDCs. Of several different markers for distinct signaling endosomes in TLR9 stimulation (14, 17, 20, 21), we targeted four markers, EEA1 for early endosome (55), VAMP3 for recycling/sorting endosomes (56, 57), LAMP2 and VAMP3 for lysosomal-related organelles (17, 21), and LAMP2 and LAMP1 for lysosomes (58). EGA treatment decreased the colocalization of CpG-ODNs with LAMP2 and LAMP1 endo-lysosomal markers in pDCs like YM201636 treatment, suggesting that TLR9 activation occurs in a LAMP^+^ compartment. However, only YM201636 could reduce CpG-ODN co-localization in VAMP3^+^ compartments. YM201636 could potentially suppress the formation of inducible VAMP3^+^LAMP2^+^ compartment in an early activation stage, as previously reported (17), but this will need further investigation in future studies.

We further demonstrated that EGA can diminish IFNα expression in SLE pDCs to TLR9 stimulation. However, EGA was not capable of diminishing TNFα expression by CpG-ODN2006/DOTAP stimulated SLE pDCs, whereas it was able to decrease TNFα expression by CpG-ODN2006/DOTAP-stimulated pDCs from healthy subjects. Whether such difference is due to the effects of any medications used for patients or one of the outcomes of altered phenotypes of SLE pDCs will need to be investigated in future studies. Although this study did not investigate mechanisms in which EGA suppress type 1 IFN and proinflammatory cytokine expression by pDCs treated with influenza virus, it was also of note that EGA could effectively suppresses both IFNα and TNFα expression by pDCs treated with influenza viruses. As TLR7 dosage has been shown to be increased in individuals with two X chromosomes due to escape from X chromosome inactivation and has also been shown to have increased localization to late endosomes/lysosomes in some SLE patients (59), further examination of whether EGA-treatment can inhibit TLR7 responses is warranted. This study also demonstrates that EGA-treatment effectively lowered IFNα expression to LL-37, an amphipathic peptide implicated in autoimmunity (1–4), to deliver nucleic acids to endo-lysosomes, suggesting that EGA might have the potential in regulating certain autoimmune disorders, including SLE. Therefore, additional studies with samples of increased numbers of patients are warranted.

In summary, this study reports that, EGA, an endosomal trafficking inhibitor, can diminish the expression of both IFNα and proinflammatory cytokine by TLR9-activated pDCs from the blood of healthy donors. Without affecting the retention of TLR9 agonists in early/recycling endosomes, EGA treatment decreased TLR9 agonists localization to late endosomes/lysosomal compartments represented by the markers LAMP1 and LAMP2 potentially accessed through the activation of Akt. More detailed mechanisms of action of EGA might also need to be further investigated. EGA was also capable to diminish IFNα expression by SLE-pDCs stimulated with TLR9 agonists. Additional avenues of research on EGA should include its utilization as a broad-spectrum agent against viral and bacterial infection, as pointed out in its initial discovery (22), as well as assessment of its potential in autoimmune disease states where TLR9 is implicated in the disease pathogenesis.

## Data availability statement

The original contributions presented in the study are included in the article/**Supplementary Material**, further inquiries can be directed to the corresponding author/s.

## Ethics statement

The studies involving human participants were reviewed and approved by Mayo Clinic Institutional Research Board. The patients/participants provided their written informed consent to participate in this study.

## Author contributions

MW - conceptualization, data curation, formal analysis, investigation, methodology, visualization, writing - original draft, and writing - review & editing. CG - investigation and writing - review & editing. HH - software and writing - review & editing. LG - data curation, investigation, resources, and writing - review & editing. MK - funding acquisition, resources, data analysis, and writing & editing. LG - funding acquisition, resources, data analysis, and writing & editing. HJ - Funding acquisition, resources, data analysis, and writing & editing. J-PG - resources and writing - review & editing. DB - methodology, resources, and writing - review & editing. SO - conceptualization, funding acquisition, project administration, supervision, writing - original draft, and writing - review & editing. All authors contributed to the article and approved the submitted version.
