# Supplementary material for: Disruption of endosomal trafficking with EGA alters TLR9 cytokine response in human plasmacytoid dendritic cells
Source: Front Immunol. 2023 Mar 20;14:1144127. doi: 10.3389/fimmu.2023.1144127 (PMC10067882; doi:10.3389/fimmu.2023.1144127)
Supplement: Supplementary file 1 [file DataSheet_1.pdf]

frontiers

Supplementary Material

Disruption of endosomal trafficking with EGA alters TLR9 cytokine response in human plasmacytoid dendritic cells

Matthew J Wiest[1,2], Chao Gu[1], Hyoungjun Ham[3], Laurent Gorvel[4], Mira T Keddis[5], Leroy W Griffing,[6], HyeMee Joo[1,2], Jean-Pierre Gorvel[7], Daniel D Billadeau[3], SangKon Oh[1, 2*]

[1]Department of Immunology, Mayo Clinic, Scottsdale, AZ, USA

[2]Baylor Institute of Biomedical Studies, Baylor University, Waco, TX, USA

[3]Department of Immunology, Mayo Clinic, Rochester, MN, USA

[4]CRCM, Aix Marseille Universite, INSERM, Marseille, France

[5]Department of Nephrology, Mayo Clinic, Scottsdale, AZ, USA

[6]Department of Rheumatology, Mayo Clinic, Scottsdale, AZ, USA

[7]CIML, Aix Marseille Universite, INSERM, Marseille, France

*** Correspondence:** SangKon Oh: Oh.Sangkon@mayo.edu

This file includes:

Supplementary Figure 1. Kinetics of EGA-mediated suppression of cytokine expression in CpG-ODN2216-stimulated pDCs.

Supplementary Figure 2. EGA diminishes IFNα and TNFα secretion in CpG-DOTAP and gDNA/LL37 stimulated pDCs.

Supplementary Figure 3. EGA and PIKfyve inhibitors reduce CpG-ODN2216 cytokine secretion in pDCs.

Supplementary Figure 4. EGA and PIKfyve inhibitors do not affect blood pDC viability.

Supplementary Figure 5. Trace of CpG-ODN2216 in plasmacytoid dendritic cells.

Supplementary Figure 6. EGA- and YM201636-treatment decrease colocalization of CpG-ODN2216-FITC with LAMP1/2$^+$ compartments.

Supplementary Figure 7. EGA and YM201636 do not affect endo-lysosomal acidification.

Supplementary Table 1. Antibodies utilized in flow cytometry in this study.

Supplementary Table 2. Antibodies utilized in immunoblotting in this study.

Supplementary Table 3. Antibodies utilized in confocal microscopy in this study.

Supplementary Table 4. Information of SLE Patients recruited in this study.

Supplementary Figure 1

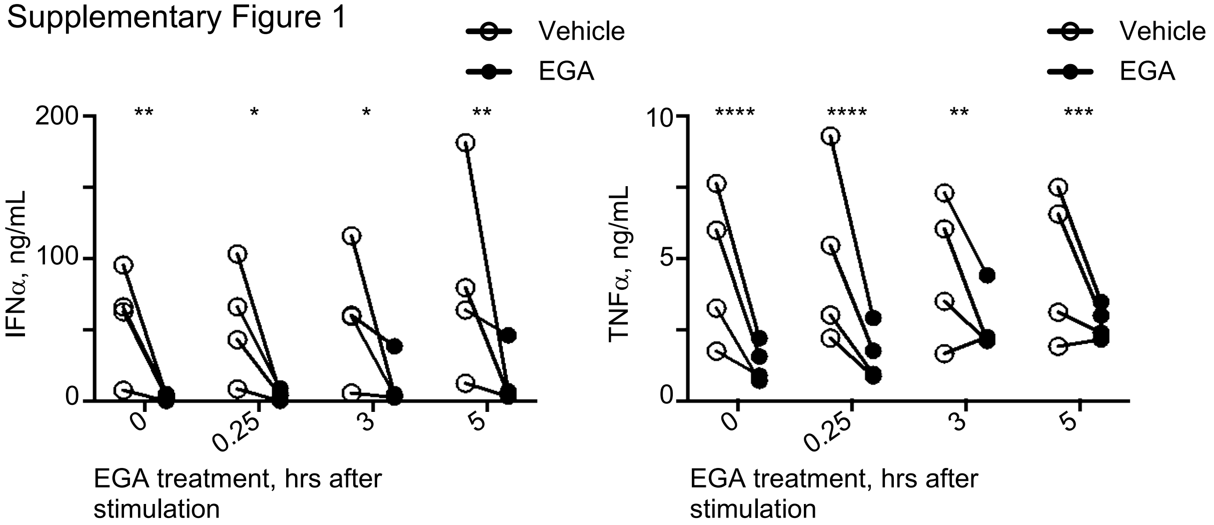

Supplementary Figure 1. Kinetics of EGA-mediated suppression of cytokine expression in CpG-ODN2216-stimulated pDCs

pDCs stimulated with CpG-ODN2216 overnight with 20 μM EGA or vehicle added at time points post-CpG-ODN2216 stimulation. IFNα and TNFα concentrations in supernatants after overnight stimulation. Data from 4 different subjects analyzed by two-way ANOVA with Sidak multiple comparison test. **P*< 0.05, ***P*< 0.01, ****P*< 0.001, *****P*<0.0001, for comparison between groups.

Supplementary Figure 2

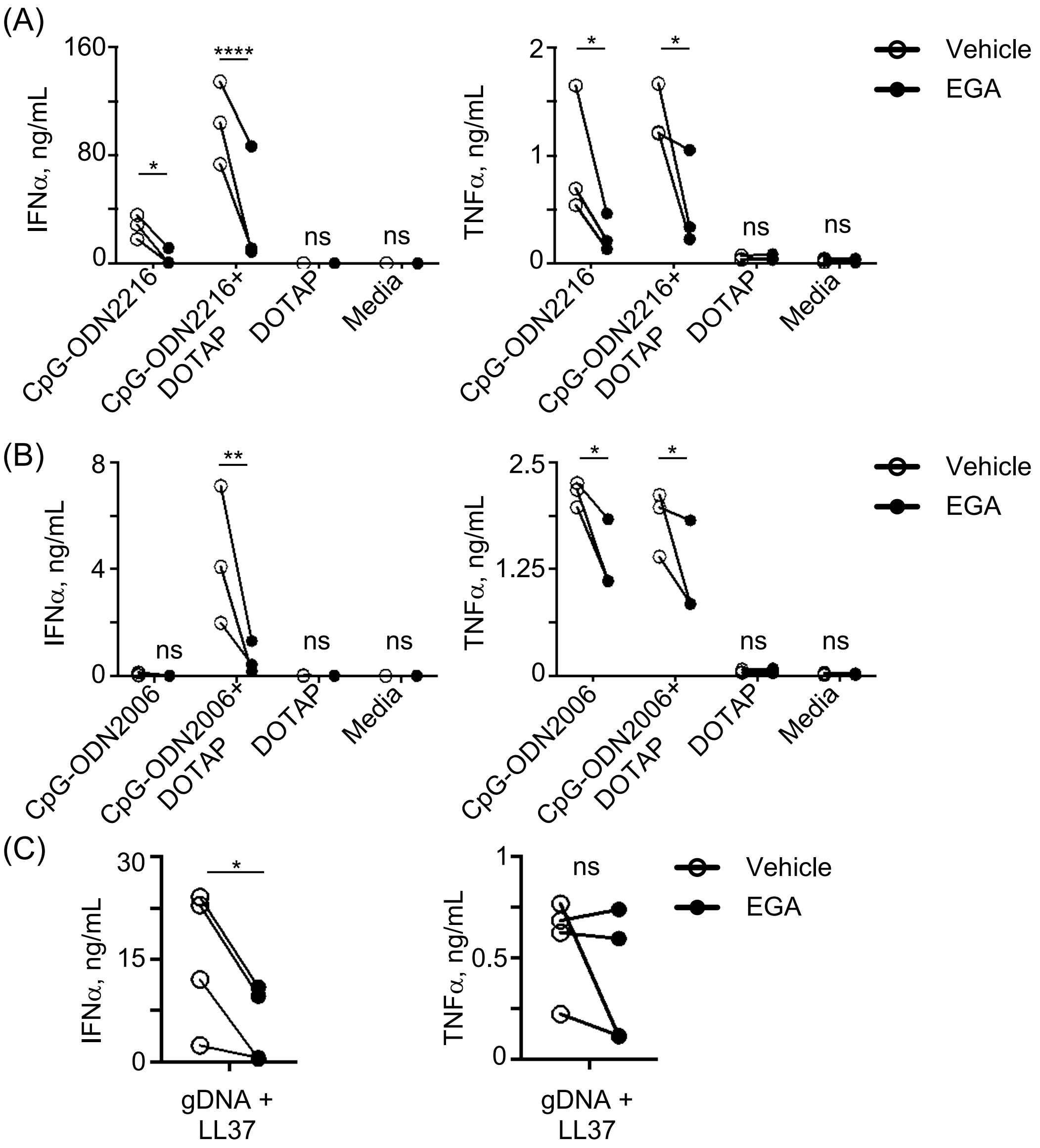

Supplementary Figure 2. EGA diminishes IFNα and TNFα secretion by CpG-DOTAP- and gDNA/LL37-stimulated pDCs.

IFNα and TNFα concentrations in supernatants of pDCs pre-treated with 20 μM EGA or vehicle and stimulated with (A) CpG-ODN2216 with/out DOTAP, (B) CpG-ODN2006 with/out DOTAP, or (C) gDNA/LL37 overnight. Data from 3-4 different subjects analyzed by two-way ANOVA with Sidak multiple comparison test (A and B) or two-tailed paired t-test (C). **P*< 0.05, ***P*< 0.01, *****P*< 0.0001, ns – not significant for comparison between groups.

Supplementary Figure 3

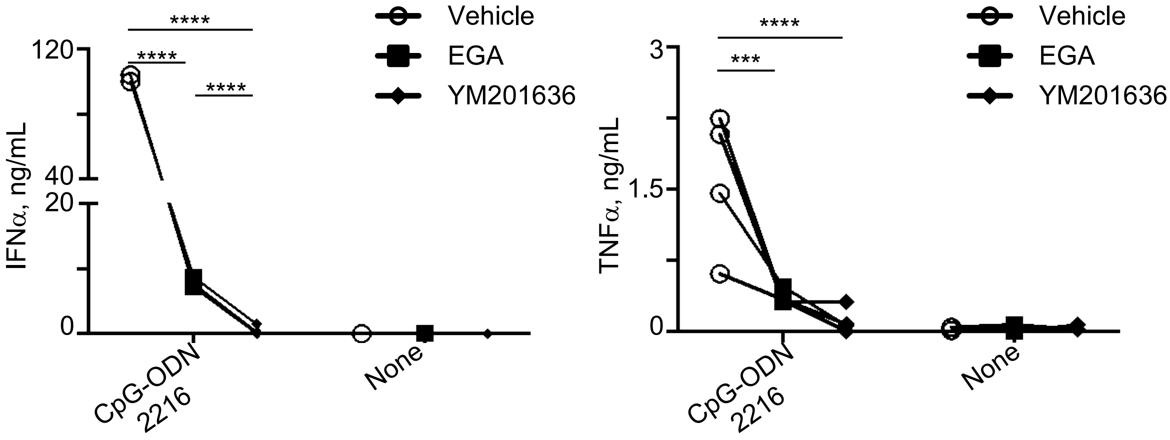

Supplementary Figure 3. EGA and PIKfyve inhibitors reduce IFNα and TNFα secretion by CpG-ODN2216-stimulated pDCs.
IFNα and TNFα levels in supernatants of pDCs pre-incubated with 20 μM EGA, 1 μM YM201636, or vehicle, and stimulated with CpG-ODN2216 overnight. Data were generated with pDCs isolated from 4 different subjects Two-way ANOVA with Tukey multiple comparison test. ***P< 0.001, ****P<0.0001, for comparison between groups.

Supplementary Figure 4

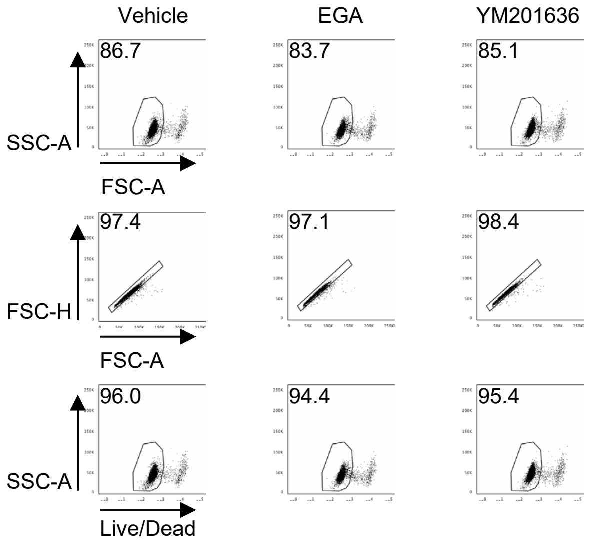

Supplementary Figure 4. EGA and PIKfyve inhibitors do not affect blood pDC viability.
Representative viability assessment of pDCs pre-incubated with 20μM EGA, 1μM YM201636, or vehicle, and stimulated with CpG-ODN2216 overnight.

Supplementary Figure 5

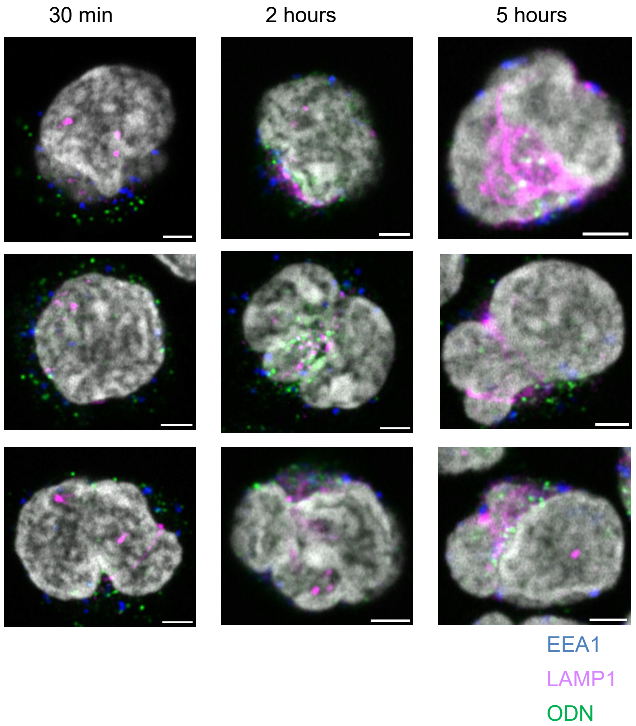

Supplementary Figure 5. Trace of CpG-ODN2216 in pDCs.
pDCs were incubated with 1μM CpG-ODN2216 – FITC. Three representative images of CpG-ODN overlap with endo-lysosomal markers (EEA1, LAMP1) at each time point. Scale bar – 2μm

Supplementary Figure 6

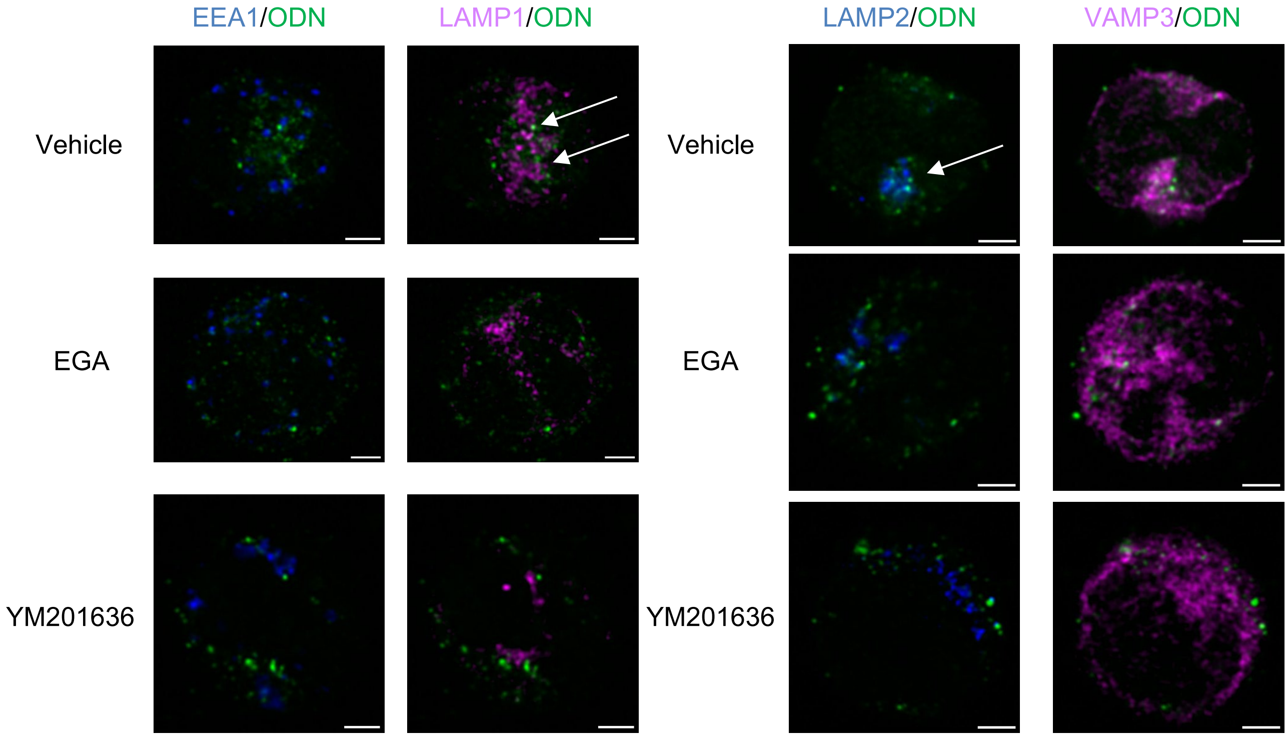

Supplementary Figure 6. EGA- and YM201636-treatment decrease colocalization of CpG-ODN2216-FITC with LAMP1/2^{+} compartments in pDCs.
pDCs were pre-incubated with 20μM EGA, 1μM YM201636, or vehicle and stimulated with 1μM CpG-ODN2216-FITC. Representative images of colocalization of CpG-ODN2216-FITC with endo-lysosomal marker proteins (EEA1, VAMP3, LAMP2, LAMP1) at 2 hours post-stimulation. Scale bar – 2μm

Supplementary Figure 7

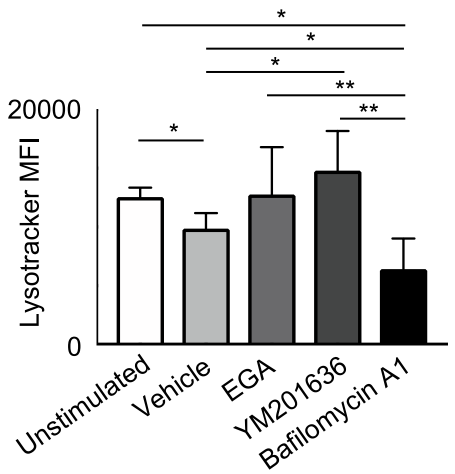

Supplementary Figure 7. EGA and YM201636 do not affect endo-lysosomal acidification.

pDCs were pre-incubated with 20 μM EGA, 1 μM YM201636, 1 μM Bafilomycin A1, or vehicle and stimulated with CpG-ODN2216 for 12 hours. CpG-ODN was washed out and cells were labeled with LysoTracker Deep Red and analyzed by flow cytometry. Data representative from 5 donors analyzed by one-way ANOVA with Tukey multiple comparison test. **P*< 0.05, ***P*< 0.01, for comparison between groups.

Supplementary Table 1. Antibodies utilized in flow cytometry in this study.

Antibody	Clone	Source	Catalog
Anti-Human HLA-DR	L243	Biolegend	307618
Anti-Human HLA-DR	G46-6	BD	560651, 561359
Anti-Human Lineage-1	Various	BD, Biolegend	340546, 363601
Anti-Human CD3	UCHT1	BD, Biolegend	555335, 300412
Anti-Human CD3	Sk7	BD	340440
Anti-Human CD14	MϕP9	BD	557831
Anti-Human CD19	HIB19	BD	555415
Anti-Human CD20	2H7	eBioscience	17-0209-42
Anti-Human CD56	B159	BD	555518
Anti-Human CD123	9F5	BD	551065, 563161
Anti-Human CD123	7G3	BD	560826
Anti-Human CD11c	B-ly6	BD	560369, 561355, 562393
Anti-Human IFNα	LT27:295	Miltenyi Biotec	130-092-601, 130-123-708
Anti-Human TNFα	Mab11	Biolegend	502932, 502936
Mouse IgG1 Isotype	MOPC-21	Biolegend	400114, 400158, 400162

Supplementary Table 2. Antibodies utilized in immunoblotting in this study.

Antibody	Clone	Catalog	Host	Source
Anti - ATF3	E9J4N	18665	Rabbit	CST
Anti - p-p65 (S536)	93H1	3033	Rabbit	CST
Anti - p65	L8F6	6956	Mouse	CST
Anti - p-IKKα/β (S176/S177)	C84E11	2078	Rabbit	CST
Anti - IKβa	44D4	4812	Rabbit	CST
Anti - p-Akt (S473)	N/A	9271	Rabbit	CST
Anti - Akt	C67E7	4691	Rabbit	CST
Anti - p-p38 (T180/Y182)	3D7	9215	Rabbit	CST
Anti - p38	N/A	9212	Rabbit	CST
Anti - pSTAT1 (Y701)	58D6	9167	Rabbit	CST
Anti - STAT1	D1K9Y	14994	Rabbit	CST
Anti - IRF7	N/A	4920	Rabbit	CST
HRP - Anti - Mouse IgG	N/A	7076	Horse	CST
HRP - Anti - Rabbit IgG	N/A	7074	Goat	CST

Supplementary Table 3. Antibodies utilized in confocal microscopy in this study.

Antibody	Clone	Catalog	Host	Source	Dilution
Anti-EEA1	14	610456	Mouse	BD	1:250
Anti-VAMP3	EPR16866	ab200657	Rabbit	Abcam	1:500
Anti-LAMP1	N/A	ab24170	Rabbit	Abcam	1:1000
Anti-LAMP2	H4B4	354301	Mouse	Biolegend	1:500
Rabbit Polyclonal IgG	N/A	LS-C149375	Rabbit	LSBio	1:5000
Mouse IgG1	MOPC-21	400102	Mouse	Biolegend	1:500
Alexa Flour 488 Goat- Anti FITC	N/A	A11096	Goat	ThermoFisher	1:1000
Alexa Flour 568-Goat-Anti-Rabbit IgG	N/A	A11036	Goat	ThermoFisher	1:1000
Alexa Flour 647-Goat-Anti-Mouse IgG	N/A	A21235	Goat	ThermoFisher	1:1000

Supplementary Table 4. Information of SLE Patients recruited in this study.

Patient	Age	Gender	Ethnicity	SLEDAI - 2k	Clinical Status
1	59	Female	White	8	2
2	39	Female	White	8	2
3	44	Female	White	12	2
4	57	Male	White	12	2
5	63	Female	White	8	2
6	37	Female	White	0	2
7	50	Male	Pacific Islander	7	2

Clinical Status

1: No disease activity, off medications

2: Disease in remission, on medication

3: Disease, relapsing

4: Treatment resistant
